# Mycoviromic Analysis Unveils Complex Virus Composition in a Hypovirulent Strain of *Sclerotinia sclerotiorum*

**DOI:** 10.3390/jof8070649

**Published:** 2022-06-21

**Authors:** Yong Wang, Zhiyong Xu, Du Hai, Huang Huang, Jiasen Cheng, Yanping Fu, Yang Lin, Daohong Jiang, Jiatao Xie

**Affiliations:** 1 State Key Laboratory of Agricultural Microbiology, Huazhong Agricultural University, Wuhan 430070, China; 13429883936@163.com (Y.W.); haidududu@163.com (D.H.); h.h@webmail.hzau.edu.cn (H.H.); jiasencheng@mail.hzau.edu.cn (J.C.); daohongjiang@mail.hzau.edu.cn (D.J.); 2 Hubei Key Laboratory of Plant Pathology, College of Plant Science and Technology, Huazhong Agricultural University, Wuhan 430070, China; yanpingfu@mail.hzau.edu.cn (Y.F.); yanglin@mail.hazu.edu.cn (Y.L.); 3 Hubei Hongshan Laboratory, Wuhan 430070, China; 4 College of Biomedicine and Health, Huazhong Agricultural University, Wuhan 430070, China; xzy@mail.hzau.edu.cn

**Keywords:** *Sclerotinia sclerotiorum*, mycovirus, co-infection, *Botourmiaviridae*, high-throughput sequencing

## Abstract

Mycoviruses are ubiquitous in pathogenic fungi including *Sclerotinia sclerotiorum*. Using RNA sequencing, more mycoviruses have been identified in individual strains, which were previously reported to be infected by a single mycovirus. A hypovirulent strain of *S. sclerotiorum*, HC025, was previously thought to harbor a single mitovirus, Sclerotinia sclerotiorum mitovirus 1 (SsMV1), based on the analysis of the conventional dsRNA extraction method. We found HC025 to be co-infected by five mycoviruses. In addition to SsMV1, four mycoviruses were identified: Sclerotinia sclerotiorum narnavirus 4 (SsNV4), Sclerotinia sclerotiorum negative-stranded RNA virus 1 (SsNSRV1), Sclerotinia sclerotiorum ourmia-like virus 14 (SsOLV14), and SsOLV22. Three mycoviruses including SsNV4, SsNSRV1, and SsOLV14 share high replicase identities (more than 95%) with the previously reported corresponding mycoviruses, and SsOLV22 shows lower identity to the known viruses. The complete genome of SsOLV22 is 3987 nt long and contains a single ORF-encoded RdRp, which shares 24.84% identity with the RNA-dependent RNA polymerase (RdRp) of Hubei narna-like virus 10 (query coverage: 26%; e-value: 8 × 10^−19^). The phylogenetic tree of RdRp suggests that SsOLV22 is a new member within the family *Botourmiaviridae*. All of the mycoviruses except for SsNSRV1 could horizontally co-transfer from HC025 to the virulent strain Ep-1PNA367 with hypovirulent phenotypes, and converted a later strain into a hypovirulent strain. In summary, we molecularly characterized the hypovirulent strain HC025 and identified five RNA mycoviruses including a new member within *Botourmiaviridae*.

## 1. Introduction

Mycovirus RNA or DNA genomes show a rich variety of taxa. According to the International Committee on Taxonomy of Viruses (ICTV), mycoviruses are mainly distributed among 26 known families [1]. Members of the family *Mitoviridae* have a monopartite, linear, positive, single-stranded RNA (+ssRNA) genome, containing only one open reading frame (ORF), which encodes the RNA-dependent RNA polymerase (RdRp). The mitoviruses reported in fungi with the shortest and longest full-length genomes are Rhizopus microsporus mitovirus 1 (accession number: LC671615, 2231 nt) and Rhizophagus diaphanum mitovirus 2 (4382 nt) [2], respectively. Furthermore, researchers have reported Heterobasidion mitovirus 3 as having a partial genome of 4955 nt [3]. Mitoviruses are thought to reside in mitochondria. Some of them have adverse effects on the hosts [4,5], whereas most have no noticeable influence [6] or unclear influences on hosts. Mycoviruses in the family *Narnaviridae* replicate in the cytoplasm and have monopartite linear +ssRNA genomes [7]. Recently, researchers have found some narnaviruses with two or more RNA segments [8]. Beauveria bassiana narna-like virus 1 is the smallest, with a length of 1689 nt [9]. *Botourmiaviridae* contains plant viruses (*Ourmiavirus* genus) and fungal viruses (*Botoulivirus*, *Scleroulivirus*, *Penoulivirus*, *Rhizoulivirus*, and *Magoulivirus* genera). Except for *Ourmiavirus*, each member of *Botourmiaviridae* has a genome with a single segment containing only one ORF encoding the RdRp [10]. Among the fungal viruses in *Botourmiaviridae,* the smallest by genome length is Botrytis cinerea ourmia-like virus 7 (2100 nt), and the longest is Botrytis cinerea ourmia-like virus 1 (5185 nt) [11]. Most viruses in *Botourmiaviridae* do not have poly(A) tails in their 3′ terminal sequences, but some such as Magnaporthe oryzae ourmia-like virus 1 do [12]. Viruses in the family *Mymonaviridae* contain a linear, negative, single-stranded RNA (-ssRNA) genome [13], and the first reported virus is Sclerotinia sclerotiorum negative-stranded RNA virus 1, which causes abnormal morphology, hypovirulence, and slow growth [14].

*S. sclerotiorum* is a worldwide necrotrophic fungal pathogen associated with more than 700 plant species, and it causes serious economic losses [15]. Hypovirulence-associated mycoviruses are potential biocontrol agents for addressing stem root rot because of their adverse effects on *S. sclerotiorum* [16,17]. Furthermore, researchers have found that a DNA virus, SsHADV-1, could impair the pathogenicity of *S. sclerotiorum* [18] and convert *S. sclerotiorum* into a beneficial endophyte that improved the yield and resistance of the plant host, rapeseed [19]. Unlike virus detection using dsRNA or DNA extraction, high-throughput sequencing technology and bioinformatics software have allowed researchers to detect and report increasing numbers of mycoviruses. Maimaiti et al. detected 151 mycovirus isolates from melon powdery mildew using RNA samples sequenced on a NextSeq500 sequencer [20]. Botella and Jung detected 15 viruses using total and small RNA-Seq in *Phytophthora condilina* [21]. Jia et al. detected 68 mycoviruses from *Sclerotinia sclerotiorum* in a single crop field [8]. In addition to detecting multiple viruses from multiple strains, researchers have commonly detected multiple mycoviruses in a single fungal strain. For example, an isolate Log1/3-8d^2^ of *Ophiostoma novo-ulmi* harbored 12 virus-like dsRNAs, three of which were determined to be related mitoviruses [22,23,24,25]. Tuomivirta and Hantula found that a single isolate SurS4 of *Gremmeniella abietina* var. *abietina* type A was infected with three mycoviruses belonging to different genera [26]. Zhu et al. characterized a complex virome in a hypovirulent *Sclerotium rolfsii* strain [27] and Mu et al. detected nine mycoviruses in a hypovirulent *S. sclerotiorum* strain SX276 [1]. We must identify the virus species in a single fungal strain to understand the biological role of a singular mycovirus.

In a previous study, Xu et al. detected one dsRNA band for the abnormal and hypovirulent *S. sclerotiorum* strain HC025 and identified it as the intermediate replication form of Sclerotinia sclerotiorum mitovirus 1 HC025 (SsMV1-HC025). Subsequently, it was verified that SsMV1-HC025 and its abnormal traits (a deformed colony morphology, a slow growth rate, a decreased number of sclerotia, and hypovirulence) could be cotransmitted from hypovirulent strains to virulent strains through hyphal fusion [28]. Therefore, we RNA-sequenced HC025 and identified and analyzed the virus species it contained. This study helped us to identify the virus species in HC025 and provided further insights into the biological function of SsMV1.

## 2. Materials and Methods

### 2.1. Strains and Cultural Conditions

The *S. sclerotiorum* strain HC025 is a hypovirulent isolate with abnormal morphology and is infected by SsMV1. The *S. sclerotiorum* strain Ep-1PNA367V is an isogenic virus-transmitted isolate of the strain Ep-1PNA367 and has a phenotype and biological properties similar to those of HC025 [28]. The *S. sclerotiorum* strain Ep-1PNA367 is a virulent isolate with normal morphology and harbors one mitovirus (Sclerotinia sclerotiorum mitovirus 6) [29]. We stored these strains on potato dextrose agar (PDA) medium slants at 4 °C and cultured them on PDA at 20 °C.

### 2.2. Total RNA Extraction and Sequencing

To prepare the sequencing sample, we cultured HC025 on a cellophane-membrane-covered PDA (CM-PDA) plate for 5 days. Then, we collected fresh mycelium, quickly froze it in liquid nitrogen, and ground it into a fine powder, which we used to extract the total RNA with the NI-*Sclerotinia sclerotiorum* RNA Extraction Reagent (NEWBIO) according to the manufacturer’s instructions.

We sent partial RNA of HC025 to a biotechnology corporation (Novogene, Beijing, China) for sequencing and stored the remaining RNA at −80 °C for subsequent virus detection. We determined the RNA integrity number (RIN) for the RNA sample using an Agilent 2100 [30] before removing the rRNA and constructing a sequencing library with a TruSeqTM RNA Sample Prep Kit (Illumina, RS-122-2001). Finally, we performed the sequencing on the Illumina MiSeq 2000/2500 platform to generate pair-end (PE) data [31].

### 2.3. Sequencing Data Analysis and Mycovirus Detection

To ensure that the subsequent analysis was reliable, we removed low-quality raw data (data with quality values less than or equal to 5% up to 40%; with N up to 10%; contaminated by the sequencing adapter) with Trimmomatic (version 0.36) [32] using the default parameter settings to obtain clean reads. 

We used HISAT2 (version 2.1.0) [33] to map the clean reads to the reference genome for *Sclerotinia sclerotiorum* 1980 UF-70 (http://fungi.ensembl.org/Sclerotinia_sclerotiorum_1980_uf_70_gca_001857865/Info/Index, accessed on 1 January 2022) and the reference mitochondrion genome for *Sclerotinia sclerotiorum* 1980 UF-70 (assembly ASM14694v2). Then, we extracted the unmapped reads, using SAMtools [34] with “-bf 12” as the parameter, which were then assembled using SPAdes (version 3.13.1) [35]. We applied CD-HIT (version 4.6.8) [36] to eliminate redundant contigs, with “-c 0.90” as a parameter.

To annotate and identify viral sequences, we analyzed the contigs using DIAMOND [37] and BLAST [38] with an e-value of 1 × 10^−5^ as the cutoff parameter. From the annotation results, we selected the contigs that had the highest matches with viruses and assembled them to merge them into longer contigs if their annotations referred to the same viruses. Finally, we selected the longest of the contigs annotated to the same virus to represent the sequence for subsequent analysis.

To detect the existence of putative mycoviruses in HC025, we used the remaining RNA sample for a reverse-transcription polymerase chain reaction (RT-PCR). To detect whether mycoviruses were transferred to Ep-1PNA367V, we extracted the total RNA of Ep-1PNA367V and subjected it to RT-PCR, as described above. The primers we used to detect the viruses are listed in Table S1.

### 2.4. Viral Sequence Extensions

We predicted the open reading frames (ORFs) for the detected viruses in the software DNAMAN (Lynnon Corporation, Quebec, Canada). However, we found that only the ORF near the 3′ end of the original contig of sequence5 was incomplete, so we designed a pair of primers (PF: TCCAAGAGAATCACTGGGTCG; PR: ATACTACCGTTGACCGGTGTC; amplicon: 406 bp) to conduct RT-PCR and Sanger sequencing to obtain the complete ORF for sequence5.

For the novel mycovirus (sequence6) we detected, we artificially divided the original contig of sequence6 into four sequentially overlapping segments. Then, we amplified each segment using the specific primers listed in Table S2. To obtain the terminal sequences, we extracted the dsRNA of HC025 cultured on CM-PDA for 5 days, as previously described [39]. Then, we ligated the RACE-OLIGO adapter to the dsRNA termini using the T4 RNA ligase (Takara). After incubating the resulting product at 4–16 °C for 24 h, we subjected it to rapid amplification of cDNA end (RACE) PCRs with specific primers (Table S2) [17].

We ligated all of the expected PCR products into the pMD18-T vector (Takara) and transformed them into the *E. coli* strain XL-10. Then, we selected the positive clones detected with specific primers for sequencing. We identified each amplicon with at least three independent clones.

### 2.5. Mycovirus Genome Characteristics and Sequence Analysis

We constructed the contig length distribution plot in RStudio [40]. We calculated the reads mapped to each mycovirus using HISAT2 and SAMtools and visualized them using IGV [41]. We calculated the viral abundance as the number of reads mapped to a sequence divided by the sequence length and visualized the results using GraphPad Prime [42]. We predicted the potential secondary structures of the viral terminal sequences using an online tool (http://rna.urmc.rochester.edu/RNAstructure.html, accessed on 8 March 2022). The percent identity matrix of viral RdRps was generated by Clustal Omega online (https://www.ebi.ac.uk/Tools/msa/clustalo/, accessed on 9 April 2022) and visualized by TBtools [43]. We conducted multiple sequence alignment using MAFFT [44] and visualized the results using Jalview [45]. Before constructing a phylogenetic tree, we removed the poorly aligned regions using trimAl [46]. Finally, we constructed a maximum-likelihood (ML) phylogenetic tree using IQ-TREE [47], with “-m MFP” and “-bb 10000” as two parameters, and visualized it using FigTree (available at http://tree.bio.ed.ac.uk/software/figtree/, accessed on 12 April 2022). 

### 2.6. Biological Properties Detection of Virus Co-Infection Strain Ep-1PNA367V

To understand the functional characterization of the co-infected viruses, we compared the growth rate, the morphology of the colony and hyphal tips, and the pathogenicity of strain Ep-1PNA367V and the isogenic strain Ep-1PNA367 referred to in the methods mentioned [28].

## 3. Results

### 3.1. Strain HC025 Is Co-Infected by Five ssRNA Mycoviruses

**Figure 1:**
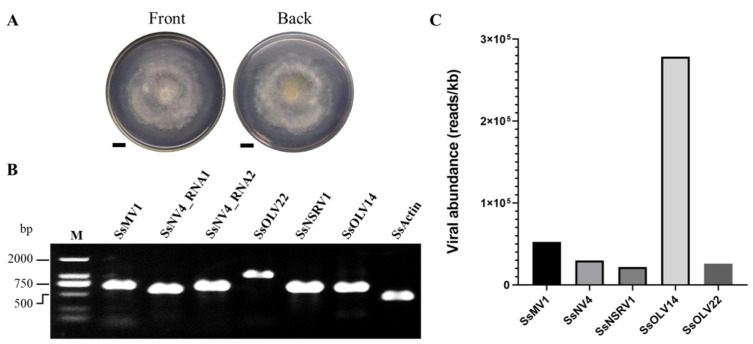
Virus detection in HC025. (**A**) The front and back of the HC025 colony morphology cultured on PDA for 6 days. The bar represents 1 cm. (**B**) The RT-PCR detection of the six viral sequences in the HC025. We used SsActin as a positive control to detect the *actin* gene of *S. sclerotiorum*. M: DL2000 DNA Marker. (**C**) The abundance analysis of the five viruses, calculated using RNA sequencing data.

The *S. sclerotiorum* strain HC025 had an abnormal colony morphology without sclerotia after growing on PDA for six days (Figure 1A), as reported in a previous study. To investigate the mycoviruses harbored in the hypovirulent strain HC025, we conducted RNA sequencing for the metatranscriptome and obtained 12.96 Gb of raw data containing 85,837,642 paired-end reads. After removing the low-quality reads, we obtained 84,182,128 clean reads (98% of the raw data) for subsequent analysis. The clean reads were mapped to the genome of *S. sclerotiorum*, and then the unmapped reads were extracted to be assembled de novo into 37,546 contigs. The length distribution range of these contigs was 89 to 10,407 nt. Six contigs were longer than 5000 nt, and the others were shorter than 5000 nt. The most abundant contigs were those shorter than 800 nt (Figure S1). 

To identify the viral sequences, we annotated the contigs using BLAST. We successfully annotated 37,248 contigs (99.2% of all the contigs), but 298 contigs could not be annotated. We extracted the contigs with annotations referring to viruses and assembled them. After filtering short sequences less than 1000 nt long and extending them with PCR, we obtained six viral sequences, which referred to five viruses: Sclerotinia sclerotiorum mitovirus 1 (SsMV1), Sclerotinia sclerotiorum narnavirus 4 (SsNV4), Sclerotinia sclerotiorum negative-stranded RNA virus 1 (SsNSRV1), Sclerotinia sclerotiorum ourmia-like virus 14 (SsOLV14), and SsOLV22 (Table 1). Three mycoviruses—SsNV4, SsNSRV1, and SsOLV14—shared high percentages of their identities (more than 95%) with the previously reported corresponding mycoviruses, whereas SsOLV22 was less similar to the known viruses, with a 24.84% shared identity. We confirmed the six viral sequences using RT-PCR and found that they existed in the HC025 strain (Figure 1B). Based on the clean sequencing reads, we calculated the abundance of each virus in HC025 and found that the most abundant virus was SsOLV14, followed by SsMV1, and the least abundant virus was SsNSRV1 (Figure 1C). We recorded all five newly detected viral sequences in the GenBank database with the accession numbers OK165495–OK165499 (Table 1).

### 3.2. Genomic Analysis of Four Newly Identified Mycoviruses Infecting HC025 Highly Similar to Known Mycoviruses

Sequence1 is 2505 nt in length, and its sequencing depth is 5821-fold (Table 1). The region from 302 to 2470 nt of sequence1 encodes a putative RNA-dependent RNA polymerase (RdRp) protein with 722 amino acids. Among the 131,476 reads mapped to sequence1, we mapped 18,898 reads to one nucleotide acid site at most, and the mapped read peaks were located in the interior region of the ORF (Figure S2). When we aligned the ORF region of sequence1 and the reported sequence of Sclerotinia sclerotiorum mitovirus 1 HC025 (KJ463570.1), we observed that they shared 99.54% of their identities. Based on these results, we suggest that sequence1 is the partial genome of SsMV1 in the HC025.

Sequence2 and sequence3 are 3031 and 2453 nt long, respectively, and their sequencing depths were 3256- and 4259-fold, respectively (Table 1). For sequence2, the region from 167 to 2920 nt encodes a putative RdRp protein with 917 amino acids. The region from 24 to 2345 nt of sequence3 encodes a putative unknown protein containing 774 amino acids. At most, we mapped 19,134 and 11,970 reads to one nucleotide acid site of sequence2 and sequence3, respectively. For sequence2, the mapped reads peaked within the end of ORF and the 3′ untranslated region (UTR), whereas the mapped reads for sequence3 peaked in the interior region of the ORF (Figure S3A). The ORFs of sequence2 and Sclerotinia sclerotiorum narnavirus 4 segment RNA1 (MW442875.1) shared 94.12%, whereas the ORFs of sequence3 and Sclerotinia sclerotiorum narnavirus 4 segment RNA2 (MW442876.1) shared 98.06% of their identities. The phylogenetic analysis for SsNV4-HC025 (Figure S3B) also supports that SsNV4-HC025 belongs to the family *Narnaviridae*.

Sequence4 is 9835 nt long, and its sequencing depth was 3203-fold (Table 1). It contains six ORFs in the regions of 178–906, 962–2143, 2240–3049, 3170–3358, 3452–9256, and 9257–9805 nt. ORFs II and V encode the nucleoprotein (N protein) and large protein (L protein), respectively. The remaining four ORFs encode hypothetical proteins. For this viral sequence, we mapped 7991 reads to it, mostly at one nucleotide acid site, and the mapped reads peaked in the interior region of each ORF (Figure S4A). The shared identities of the six putative proteins of sequence4 and the corresponding putative proteins of Sclerotinia sclerotiorum negative-stranded RNA virus 1-WX (MT646384.1) were 97.11%, 98.93%, 96.28%, 95.16%, 98.50%, and 92.13%. The six ORFs in sequence4 shared 94.38%, 95.66%, 94.07%, 93.65%, 95.69%, and 93.44% of their identities with those of Sclerotinia sclerotiorum negative-stranded RNA virus 1-WX (MT646384.1). The phylogenetic analysis for sequence4 based on the L proteins (Figure S4B) also supports that it belongs to the genus *Sclerotimonavirus* of the family *Mymonaviridae*. Based on these results, we believe that we identified mycovirus Sclerotinia sclerotiorum negative-stranded RNA virus 1 in HC025.

Sequence5 is 1913 nt long, and its sequencing depth was 7391-fold (Table 1). The region from 33 to 1796 nt encodes a putative RdRp protein with 587 amino acids. At most, we mapped 175,361 reads to one nucleotide acid site of sequence5. Of the two mapped read peaks, one was entirely in the interior region and the other partially covered the 3′ UTR region (Figure S5A). The ORF region shared 96.43% of its identity with Sclerotinia sclerotiorum ourmia-like virus 14 (MT646410.1). The phylogenetic analysis based on RdRp revealed that SsOLV14-HC025 belongs to *Botourmiaviridae* (Figure S5B). Thus, we identified Sclerotinia sclerotiorum ourmia-like virus 14 in HC025.

### 3.3. A Novel Virus Related to Members of the Family Botourmiaviridae

**Figure 2:**
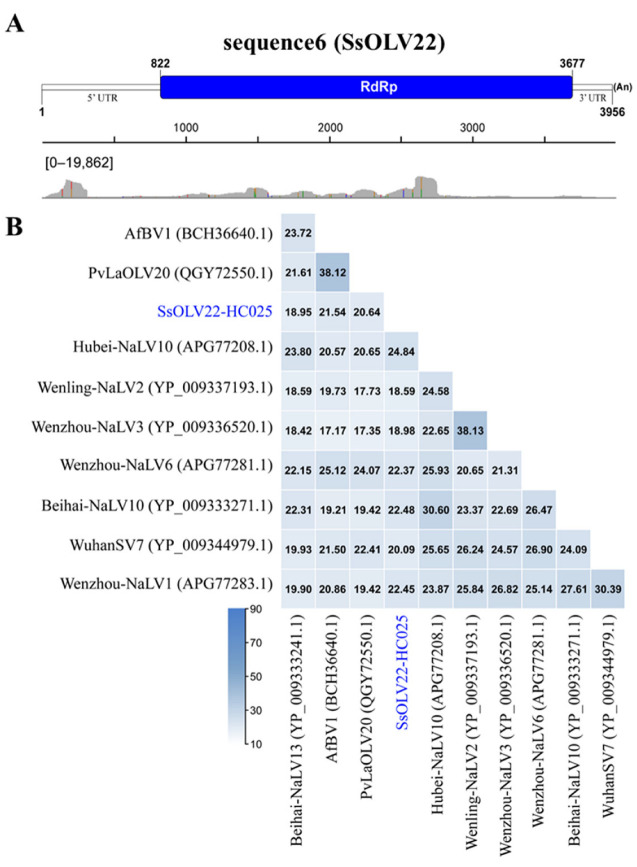
The viral identity analysis and genome structure of virus SsOLV22. (**A**) Schematic organization and annotation of the SsOLV22-HC025 genome. The putative ORF is indicated as a box. “(An)” represents the poly(A) tail. The read distribution profile for sequence6 is demonstrated below. The number in the square bracket indicates the number range of reads mapped to each nucleotide acid site in the sequence. (**B**) Percent identity matrix of viral RdRp sequences. AfBV1: Aspergillus fumigatus botourmiavirus 1; PvLaOLV20: Plasmopara viticola lesion associated ourmia-like virus 20; Hubei-NaLV10: Hubei narna-like virus 10; Wenling-NaLV2: Wenling narna-like virus 2; Wenzhou-NaLV3: Wenzhou narna-like virus 3; Wenzhou-NaLV6: Wenzhou narna-like virus 6; Beihai-NaLV10: Beihai narna-like virus 10; WuhanSV7: Wuhan spider virus 7; Wenzhou-NaLV1: Wenzhou narna-like virus 1; Beihai-NaLV13: Beihai narna-like virus 13.

**Figure 3:**
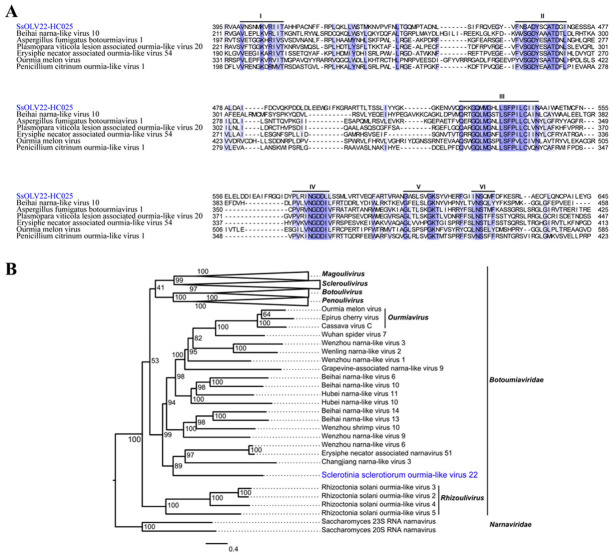
The multiple alignment and phylogenetic analysis of SsOLV22-HC025. (**A**) Multiple alignment of SsOLV22-HC025 and its highest matched viruses based on BLASTX from the NCBI. (**B**) The phylogenetic analysis of SsOLV22-HC025. This maximum-likelihood phylogenetic tree was constructed based on the amino acid sequences of viral RdRps. SsOLV22-HC025 is marked in blue.

Sequence6 is 3987 nt in length, has a GC content of 41% including a poly(A) tail of 31 nucleotides, and is a fully viral cDNA genome. The 5′ UTR is 821 nt, and the 3′ UTR is 279 nt, excluding the poly(A) tail. The viral genome has a putative ORF (822–3677 nt; initiation codon: AUG; termination codon: UAG) encoding an RdRp with 951 amino acids (110 kDa) using a universal codon or mitochondrial codon. Its sequencing depth was 2991-fold (Table 1). We mapped 19,862 reads to mostly one nucleotide acid site of sequence6. Most of the mapped reads concentrated in the ORF region, whereas a prominent mapped read peak occurred at approximately the 5′ terminal from 25 to 313 nt (Figure 2A). BLASTp alignment of its putative RdRp indicated that sequence6 shared the highest identity (24.84%) with Hubei narna-like virus 10 (query coverage: 26%; e-value: 8 × 10^−19^), which was followed by Beihai narna-like virus 10 (query coverage: 43%; e-value: 3 × 10^−22^; accession: YP_009333271.1) with an amino acid identity of 22.48% (Figure 2B). We also conducted multiple alignment of the RdRps of sequence6 and other relative viruses. We detected six conserved motifs including the GDD motif (Figure 3A). Based on the RdRp, we phylogenetically analyzed sequence6 and found that it was a member of the family *Botourmiaviridae* outside of other definite or proposed genera (Figure 3B). Based on these results, we believe that we identified a novel mycovirus, which we designated as Sclerotinia sclerotiorum ourmia-like virus 22-HC025 (SsOLV22-HC025).

**Figure 4:**
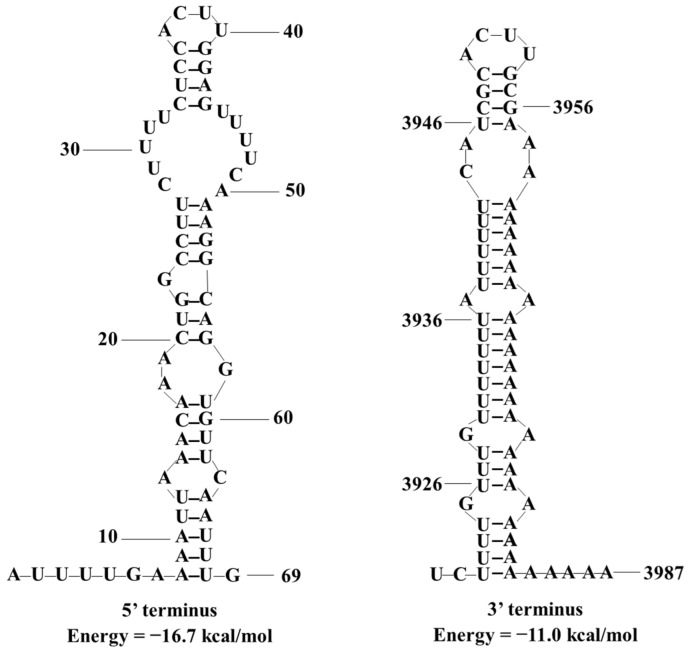
The predicted secondary RNA structure of the 5′ and 3′ terminal sequences of SsOLV22-HC025. We predicted and calculated the potential stem–loop structures and the corresponding free energy on the online RNAstructure web servers.

We predicted the terminal RNA structures of SsOLV22-HC025 online and found that the 5′ terminal region (1–69 nt) and the 3′ terminal region (3919–3987 nt) could fold in stem–loop structures with free energies of −16.7 and −11.0 kcal/mol, respectively (Figure 4). However, the 5′ and 3′ terminal sequences failed to form a reverse complementary panhandle structure.

### 3.4. Four Mycoviruses Can Transfer Horizontally via Hyphal Fusion

**Figure 5:**
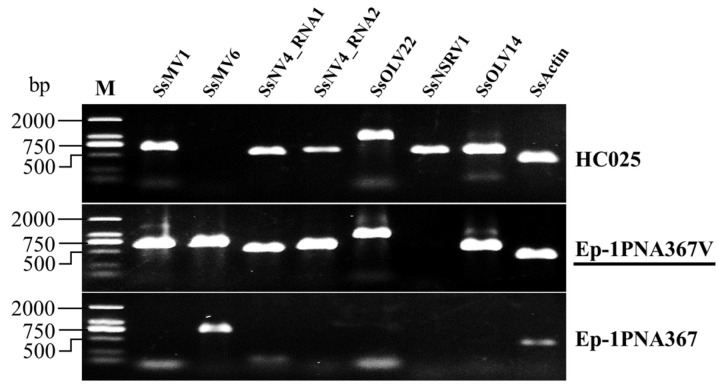
Virus detection in Ep-1PNA367V. SsMV6 refers to the primers for detecting Sclerotinia sclerotiorum mitovirus 6; SsActin refers to the primers for detecting the *actin* gene of Sclerotinia sclerotiorum. Ep-1PNA367V and Ep-1PNA367 are isogenic strains, and Ep-1PNA367V was derived from the dual culture of strains HC025 and Ep-1PNA367. M: DL2000 DNA Marker.

Considering the above results, we wondered whether Ep-1PNA367V could be infected by multiple viruses from HC025 instead of the singular reported mycovirus (SsMV1). From the RT-PCR results, we deduced that Ep-1PNA367V was infected by SsMV1 (as reported in previous research); Sclerotinia sclerotiorum mitovirus 6 (SsMV6), which existed in the original Ep-1PNA367 strain; SsNV4; SsOLV22; and SsOLV14 (Figure 5). Thus, we believe that Ep-1PNA367V is infected by multiple, positive, single-stranded RNA viruses.

### 3.5. Virus Co-Infection Endows Abnormal Biological Characteristics to S. sclerotiorum

**Figure 6:**
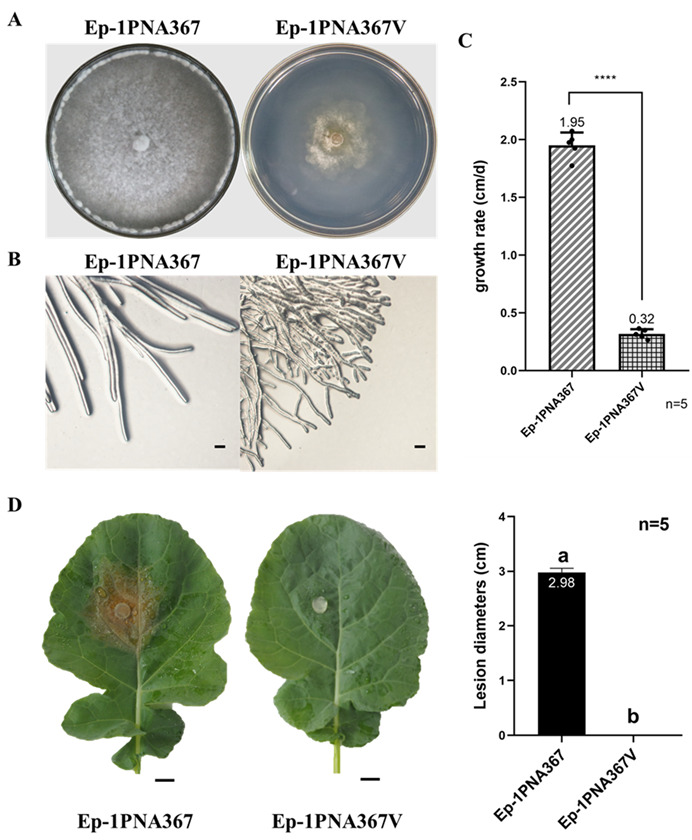
The biological characteristics detection of strain Ep-1PNA367V. (**A**) Colony morphology of strains Ep-1PNA367 and Ep-1PNA367V on PDA was observed at 7 days post-inoculation (dpi). (**B**) Hyphal tips of the two strains observed at 3 dpi. The bar refers to 100 μm. (**C**) Growth rate detection. The stars refer to significantly difference between strains Ep-1PNA367 and Ep-1PNA367V. (**D**) Pathogenicity detection on rapeseed leaves at 2 dpi. The bar refers to 1 cm.

To understand the effects of the four +ssRNA viruses on S. sclerotiorum, we detected the biological characteristics of the strain Ep-1PNA367V. The strain Ep-1PNA367V had an abnormal colony morphology (Figure 6A) similar to that of strain HC025 (Figure 1A). The hyphal tips of strain Ep-1PNA367V became thin and multi-branched compared to the isogenic strain Ep-1PNA367 (Figure 6B). We found that the strain Ep-1PNA367V grew significantly slower than strain Ep-1PNA367 (Figure 6C) and could not form obvious lesions on detached rapeseed leaves (Figure 6D). This suggests that the co-infection of the four +ssRNA viruses is related to the deformity, growth, and pathogenicity reduction in *S. sclerotiorum*.

## 4. Discussion

In this study, we characterized the viruses in the hypovirulent *S. sclerotiorum* strain HC025 through the high-throughput sequencing of its total RNA. We identified five mycoviruses including the previously reported SsMV1, three newly found mycoviruses described by other researchers [8,14], and a novel botourmiavirus named SsOLV22-HC025. These detected viruses belong to four families (*Mitoviridae, Narnaviridae, Botourmiaviridae,* and *Mymonaviridae*), suggesting that HC025 has a complex mycovirome. 

The sequencing and analysis methods we used to detect the viruses were likely to have been reliable because we detected SsMV1-HC025 with a nearly complete genome and high identity match with the originally reported sequence. However, differences between SsMV1-HC025 and the original sequence remain. Several reasons may explain this. Mitoviruses enter the host mitochondria, which are the main ROS-production sites [48,49]. An accumulation of ROS can damage the biomolecules [50]. Under ROS pressure, mutations may occur and accumulate in the viral genome. Although Illumina sequencing has a high accuracy rate, substitutions and indel errors are other possible explanations [51].

Among the five detected viruses, we mapped the highest number of reads to the SsOLV14-HC025 genome (Table 1), and SsOLV14 showed the highest viral abundance (Figure 1C). Researchers in a previous study observed only one clear segment when conducting electrophoresis of HC025 dsRNA, which they identified as the intermediate replication form of the virus SsMV1 [28]. Considering their results, we speculate that the SsOLV14 content was originally the highest. However, its replication activity is relatively weak, so the researchers could not detect as much replication intermediate using the dsRNA extraction method.

We detected a novel virus, SsOLV22-HC025, with a complete genome in HC025. We checked the 21 reported botourmiaviruses infecting *S. sclerotiorum* according to the NCBI and found that SsOLV22-HC025 had the longest genome and was the only virus with a poly(A) tail in its 3′ end. Only three of the botourmiavirus sequences detected in fungi have poly(A) tails. Magnaporthe oryzae botourmiavirus 9 from strain SH05 contains a 9 nt poly(A) tail [52]; Armillaria mellea ourmia-like virus 1 from strain CMW50256 contains a 7 nt poly(A) tail [53]; and Magnaporthe oryzae botourmiavirus 6 from strain YC81-2 contains a 7 nt poly(A) tail [54]. Differing from them, SsOLV22-HC025 has a long poly(A) tail of 31 nt. Furthermore, in the phylogenetic tree, SsOLV22-HC025 is close to many narna-like viruses and the genus *Ourmiavirus* (Figure 3B). Thus, we believe that SsOLV22-HC025 is different from other classified fungal botourmiaviruses and has a unique evolutionary position.

According to our results, HC025 is a multiple-virus-infected strain. Researchers have only studied SsNSRV1 for its hypovirulence-associated function [14,55]; the other four mycoviruses have not been separately functionally researched. As shown in Figure 5 and Figure 6, the virus-transmitted strain Ep-1PNA367V obtained all the viruses detected in HC025 except for the hypovirulence-associated virus SsNSRV1, and became abnormal with reduced growth rate and pathogenicity. This suggests that SsMV1-HC025, SsNV4-HC025, SsOLV14-HC025, and SsOLV22-HC025 can transfer together, and the decay traits are related with the co-infection of these four +ssRNA viruses. In a previous study on HC025, the vast majority of the mitochondria in Ep-1PNA367V were abnormal. Mitochondria are essential organelles and have a crucial impact on cell health [56,57]. Therefore, it was regarded that SsMV1-HC025 was the hypovirulence-associated factor. When analyzing the sequences of SsOLV14-HC025 and SsOLV22-HC025, we found that they could all encode RdRps of the same length, whether using a universal or mitochondrial codon. This implies that these two viruses can also replicate in host mitochondria. We speculate that they also affect mitochondrial function. The genome of SsNV4-HC025 has two segments, with the RNA1 encoding RdRp, but RNA2 encodes an unknown protein [8] that may interact with its host. To determine which mycovirus is responsible for hypovirulence, the transfection of each virus with viral RNA synthesized in vitro should be conducted. Alternatively, we could study the four +ssRNA viruses as one “virus group” and focus on the influence of *S. sclerotiorum* at the molecular level such as its effects on genes, proteins, or metabolites to find essential targets for controlling stem and root rot.

## 5. Conclusions

We discovered that the *S. sclerotiorum* strain HC025 was co-infected with five mycoviruses and obtained the full-length genome of a novel botourmiavirus, SsOLV22. The abnormal biological traits of HC025 are related with the co-infection of the four +ssRNA viruses. Researchers must conduct further studies to illuminate the biological functions of each virus in strain decay and hypovirulence.

## Figures and Tables

**Table 1:** The viruses in HC025.

Name	Length (nt)	Name of Putative Virus	Best Match	Identity (aa, %)	Reads	Accession Number
sequence1	2505	SsMV1	Sclerotinia sclerotiorum mitovirus 1 (YP_009121785.1)	95.32	131,476	KJ463570
sequence2	3031	SsNV4_RNA1	Sclerotinia sclerotiorum narnavirus 4 RNA1 (QZE12024.1)	96.35	87,059	OK165495
sequence3	2453	SsNV4_RNA2	Sclerotinia sclerotiorum narnavirus 4 RNA2 (QZE12025.1)	98.85	76,859	OK165496
sequence4	9835	SsNSRV1	Sclerotinia sclerotiorum negative-stranded RNA virus 1-WX (QUE49133.1)	98.5	214,467	OK165497
sequence5	1913	SsOLV14	Sclerotinia sclerotiorum ourmia-like virus 14 (QUE49177.1)	98.16	533,197	OK165498
sequence6	3987	SsOLV22	Hubei narna-like virus 10 (APG77208.1)	24.84	103,130	OK165499

## Data Availability

The sequences reported in the present manuscript have been deposited in the GenBank database under accession numbers OK165495–OK165499.

## Supplementary Materials

The following supporting information can be downloaded at: https://www.mdpi.com/article/10.3390/jof8070649/s1, Table S1: Primers for virus detection in this paper, Table S2: Primers for full-length genome confirmation of SsOLV22, Figure S1: Contig length distribution histogram, Figure S2: Genome analysis of SsMV1 in strain HC025, Figure S3: Genome and phylogenetic analysis of SsNV4 in strain HC025, Figure S4: Genome and phylogenetic analysis of SsNSRV1 in strain HC025, Figure S5: Genome and phylogenetic analysis of SsOLV14 in strain HC025.
