# Supplementary material for: Mycoviromic Analysis Unveils Complex Virus Composition in a Hypovirulent Strain of Sclerotinia sclerotiorum
Source: J Fungi (Basel). 2022 Jun 21;8(7):649. doi: 10.3390/jof8070649 (PMC9317179; doi:10.3390/jof8070649)
Supplement: Supplementary file 1 [file jof-08-00649-s001.zip › jof-1762146-supplementary.pdf]

Journal of
Fungi

MDPI

Supplementary Materials

Table S1. Primers for virus detection in this paper.

Name	Target	Primer name	Primer sequence	Amplicon size (bp)
SsMV1	sequence1	F	CACCGAGCATTCCATACTGG	671
		R	AGTCCAGAGATCTCAACGCC	
SsNV4_RNA1	sequence2	F	TTCCATCCAGTTGAGGCAGC	542
		R	GATTCCAAGACGTCCCATGG	
SsNV4_RNA2	sequence3	F	TTCTTGTCCGAGATCGAACAGG	634
		R	TCTTCTCTTTGGCTGGTCCAG	
SsNSRV1	sequence4	F	CGTTGGCGATGATGTATTACGC	610
		R	GTTGCTGCAACATCGAAGGAC	
SsOLV14	sequence5	F	GAACAGACCTCATGGTGCCT	606
		R	TGCTCGACTTCGAAACCGAC	
SsOLV22	sequence6	F	GATTTCTCAGGCTCAGCGC	1013
		R	GTACTTTCCTTCTCCGGACGC	
SsMV6	-	F	GCTATTACAGATGCGGTAACGC	693
		R	ACGTAACAGAGATGGAACTCCG	
SsActin	-	F	GCACCAGAGGAGCACCCAGTTTT	415
		R	TGGAAGATTGACTGGCGGTTTGA	

SsMV6 refers to the primes for the detection of Sclerotinia sclerotiorum mitovirus 6; SsActin refers to the primers for the detection of the *actin* gene of *Sclerotinia sclerotiorum*.

Table S2. Primers for full-length genome confirmation of SsOLV22.

Primer	Sequence	Primers used for
SsOLV22P1F	AGCCTTCTTTTCTCCACTTGGAG	
SsOLV22P1R	CTCTTCTCTCGTGCTTCTGCG	
SsOLV22-HC025-det1F	GATTTCTCAGGCTCAGCGC	
SsOLV22-HC025-det1R	GTACTTTCCTTCTCCGGACGC	determination of internal sequence
SsOLV22P2F	CGGCTGCTTATGGCAGTATG	
SsOLV22P2R	AGGAAACACTCCGCTAGACG	
SsOLV22P3F	TTGATCCGCCTCTTCCCAGAAG	
SsOLV22P3R	ACTTGGACTTCCGTGCAGTG	
SsOLV22end3P1	CTTGAGAGATCGCCCACTTGC	
SsOLV22end3P2	CAATGCCGACTCTTTAGGCAG	
SsOLV22end5P1	CTTACCGCACATGAAAACTC	
SsOLV22end5P2	TTTGGTAGAGAGTAGCCACA	
RACE-OLIGO	GCATTGCATCATGATCGATCGAATTCT TTAGTGAGGGTTAATTGCC-(NH2)	determination of terminal sequence
O5RACE-1	GGCAATTAACCCTCACTAAAG	
O5RACE-2	TCACTAAAGAATTCGATCGATC	
O5RACE-3	CGATCGATCATGATGCAATGC	

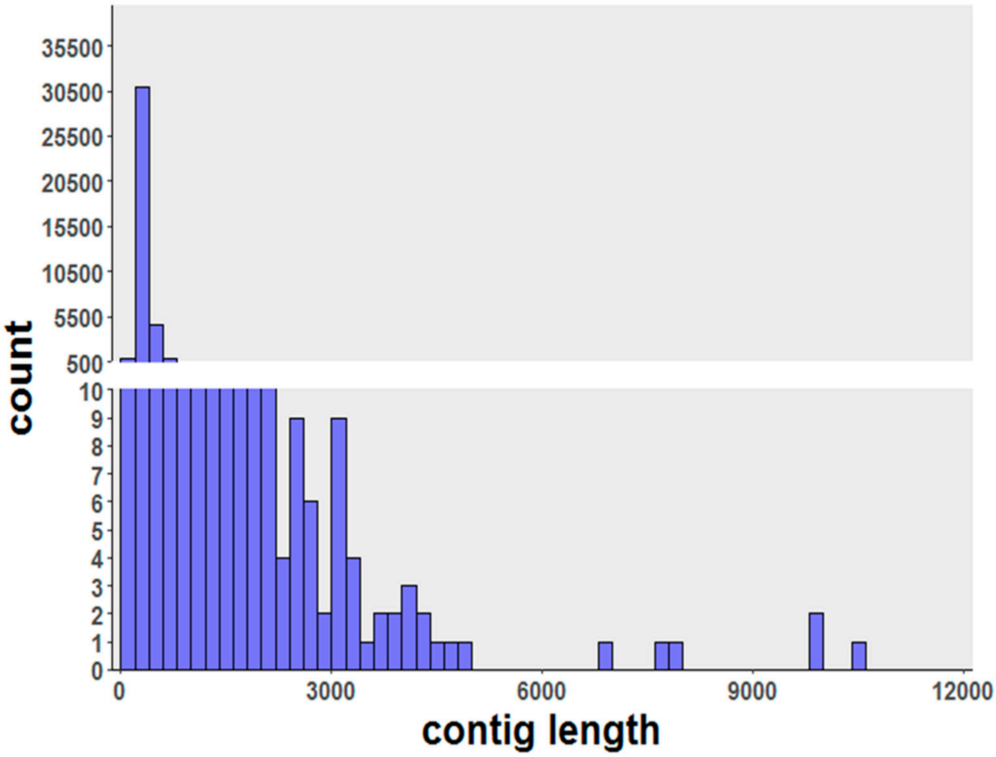

Figure S1. Contig length distribution histogram.

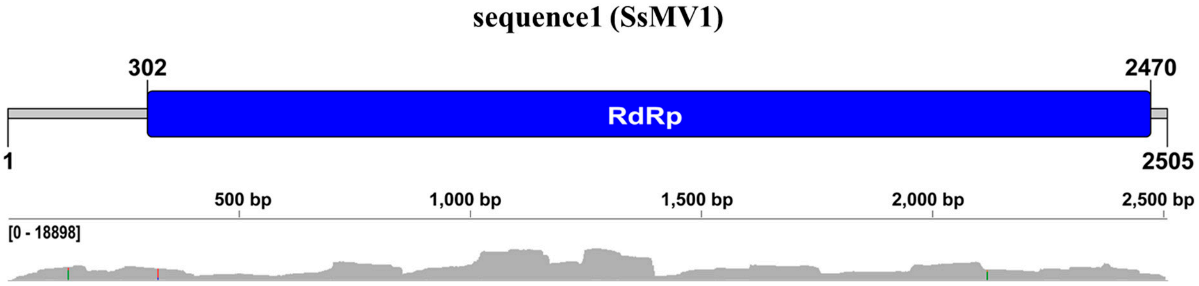

Figure S2. Genome analysis of SsMV1 in strain HC025. Schematic organization and annotation of the genome of SsMV1. The putative ORF was indicated as a (upper panel), and the reads distribution profile was demonstrated below. The number in the square bracket indicated the number range of reads mapped to each nucleotide acid site of the sequence.

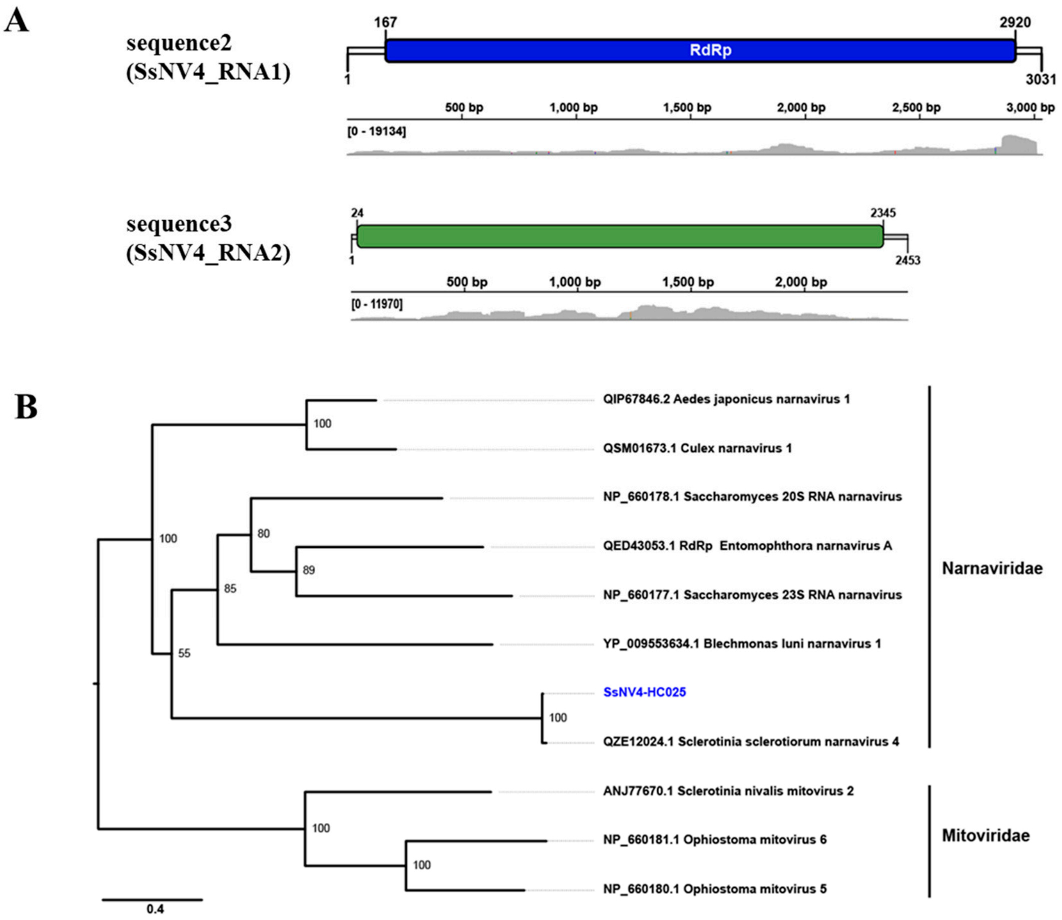

Figure S3. Genome and phylogenetic analysis of SsNV4 in strain HC025. (**A**) Schematic organization and annotations of the genome of SsNV4-HC025. The putative ORFs of each segment were indicated as boxes, while the reads distribution profile was demonstrated below. The number in the square bracket indicated the number range of reads mapped to each nucleotide acid site of the sequences. (**B**) The phylogenetic analysis of virus SsNV4-HC025. This maximum likelihood phylogenetic tree was constructed based on the amino acid sequences of viral RdRps. Virus SsNV4-HC025 was marked with blue colour.

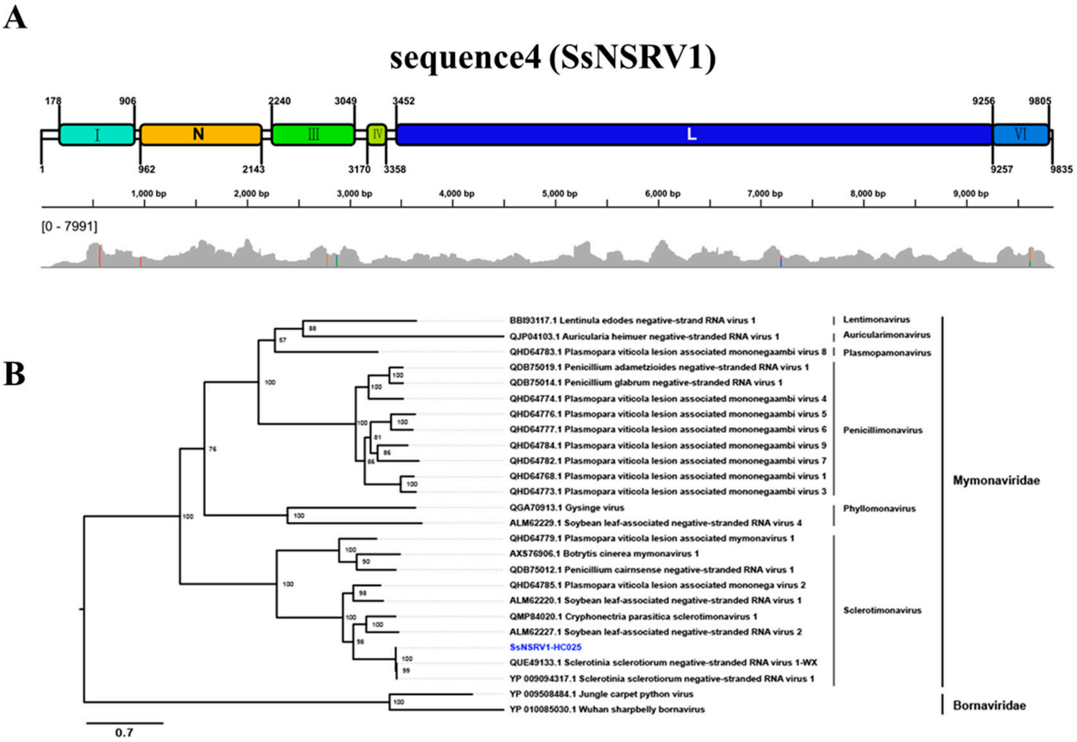

Figure S4. Genome and phylogenetic analysis of SsNSRV1 in strain HC025. (**A**) Schematic organization and annotation of the SsNSRV1-HC025 genome. The putative ORFs were indicated as boxes of various lengths (upper panel), and the reads distribution profile was demonstrated below. N refers to nucleoprotein and L refers to large polymerase. The number in the square bracket indicated the number range of reads mapped to each nucleotide acid site of the sequence. (**B**) The phylogenetic analysis of the virus SsNSRV1-HC025. This maximum likelihood phylogenetic tree was constructed based on the amino acid sequences of viral L protein. Virus SsNSRV1-HC025 was marked with blue colour.

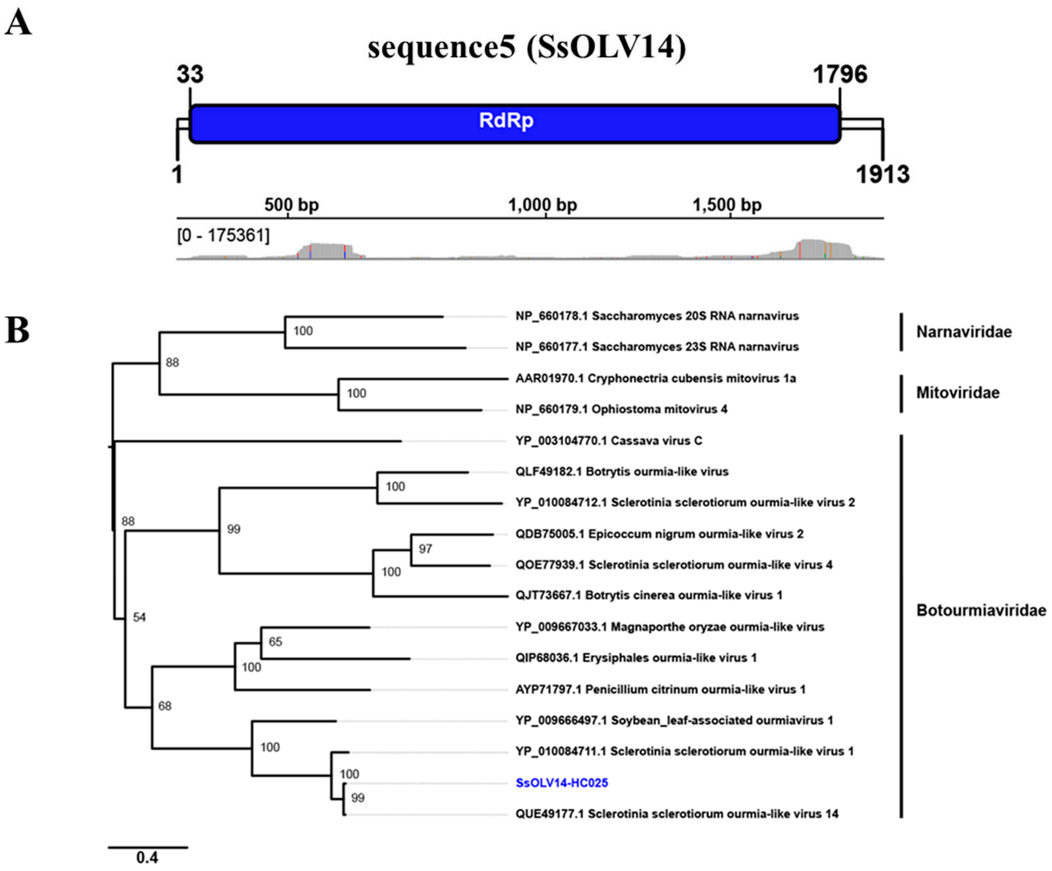

Figure S5. Genome and phylogenetic analysis of SsOLV14 in strain HC025. (**A**) Schematic organization and annotation of the SsOLV14-HC025 genome. The putative ORF was indicated as a box. The reads distribution profile of the sequence was demonstrated below. The number in the square bracket indicated the number range of reads mapped to each nucleotide acid site of the sequence. (**B**) the phylogenetic analysis of the virus SsOLV14-HC025. This maximum likelihood phylogenetic tree was constructed based on the amino acid sequences of viral RdRps. Virus SsOLV14-HC025 was marked with blue colour.
